# Comparing co-morbidities in total joint arthroplasty patients using the RxRisk-V, Elixhauser, and Charlson Measures: a cross-sectional evaluation

**DOI:** 10.1186/s12891-015-0835-4

**Published:** 2015-12-10

**Authors:** Maria C. S. Inacio, Nicole L. Pratt, Elizabeth E. Roughead, Stephen E. Graves

**Affiliations:** Quality Use of Medicines and Pharmacy Research Centre, Medicine and Device Surveillance Centre of Research Excellence, Sansom Institute, School of Pharmacy and Medical Sciences, University of South Australia, GPO Box 2471, Adelaide, 5001 South Australia Australia; Australian Orthopaedic Association, National Total Joint Replacement Registry, Level 6 Bice Building, Royal Adelaide Hospital, The University of Adelaide, Adelaide, 5005 SA Australia

**Keywords:** Co-morbidities, Total joint arthroplasty, Pharmacy data, RxRisk-V, Charlson, Elixhauser

## Abstract

**Background:**

Joint arthroplasty patients have a high prevalence of co-morbidities and this impacts their surgical outcomes. There are different ways to ascertain co-morbidities and appropriate measurement is necessary. The purpose of this study was to: (1) describe the prevalence of co-morbidities in a cohort of total hip arthroplasty (THA) and knee arthroplasty (TKA) patients using two diagnoses-based measures (Charlson and Elixhauser) and one prescription-based measure (RxRisk-V); (2) compare the agreement of co-morbidities amongst the measures.

**Methods:**

A cross-sectional study of Australian veterans undergoing THAs (*n* = 11,848) and TKAs (*n* = 18,972) between 2001 and 2012 was conducted. Seventeen co-morbidities were identified using the Charlson, 30 using the Elixhauser, and 42 using the RxRisk-V measure. Agreement between co-morbidities was calculated using Kappa (κ) statistics.

**Results:**

Combining measures, 64 conditions were identified, of these 28 were only identified using the RxRisk-V, 11 using the Elixhauser, and 2 using the Charlson. The most prevalent conditions was pain treated with anti-inflammatories (58.7 % THAs, 55.9 % TKAs), pain treated with narcotics (55.0 % THAs, 50.9 % TKAs), hypertension (56.0 % THAs and TKAs), and anticoagulation disorders (53.0 % THAs, 48.6 % TKAs). Diabetes was the only condition with substantial agreement (all κ > 0.6) amongst all measures. When comparing the diagnoses based algorithms, agreement was high for overlapping conditions (all κ > 0.71).

**Conclusions:**

Different measures identified different co-morbidities, provided different estimates for the same co-morbidity, and had different levels of agreement for common co-morbidities. This highlights the importance of understanding co-morbidity measures and using them appropriately in studies and case-mix adjustments analyses.

## Background

The prevalence of multi-morbidity is increasing [1, 2], thus the number of patients with multiple co-morbidities undergoing total joint arthroplasty has also increased in the last two decades [3, 4]. Studies in the United States (US) report multiple co-morbidities in 62 % of patients over 65 years old and this was associated with higher healthcare utilisation, cost, and overall impairment of patients [2]. In the US in the mid-2000s, patients older than 65 years undergoing total joint arthroplasty had an average of two co-morbidities when measured by the diagnosis based Elixhauser co-morbidity measure [3, 4]. The number of co-morbidities as well as specific conditions are related to patients’ surgical outcome during total joint arthroplasty [5–12].

Appropriate measurement of patient co-morbidities in those undergoing joint arthroplasty is critical because of the high prevalence of co-morbidities, the higher service utilisation, and the poorer surgical outcomes in those patients. Several co-morbidity measures exist [13–15]. The validated and most frequently used diagnostic based coding measure have been shown to be predictive of several healthcare outcomes in different study populations (including orthopaedics and other disciplines) and are used in case mix adjustment [15, 16]. Two of the most commonly used co-morbidity measures in health services and orthopaedics research are the Charlson [17] and Elixhauser [18] measures. Both measures have been used in studies evaluating total joint arthroplasty patients. Prescription medication based co-morbidity measures, such as the RxRisk-V [19] and Medication Based Disease Burden Index [20], were also developed to estimate the prevalence of co-morbidities in different populations and have been used in case mix adjustment and outcome prediction. However, to our knowledge, no previous study has evaluated the prevalence of co-morbidities in a total joint arthroplasty sample using a validated prescription-based coding measure.

Each method to ascertain co-morbidities has different strengths and limitations. For example, pharmacy based measures may provide more detailed information on active chronic conditions for which patients are actually receiving care and not only serious conditions that diagnostic based measures may identify. Further, difference in coding practices between countries may make one measure more relevant in a certain country. In Australia, for example, the use of the diagnoses based measures is limited to inpatient encounters as outpatient encounters are not recorded using the same coding system necessary for identification of the conditions. Additionally, in Australia and the US, hospital coding does not have an official mechanism to include diagnoses other than those relevant to the hospital stay [21]. This is probably avoided by countries such as the United Kingdom, which implemented in 2010 a list of required comorbidities that must be reported at the time of hospitalisation [15].

It is clinically and scientifically important to have an accurate, comprehensive, and standardized method to identify the presence of co-morbidities at the time of surgery for several reasons. One reason is to inform surgeons, healthcare workers, and patients on the proper course of treatment and risk of procedure, especially when multiple co-morbidities are present. Another is for standardizing research, specifically when evaluating outcomes in different cohorts of patients where proper case mix adjustment must be conducted to assure cohorts can be properly compared. Our study sought to (1) determine the prevalence of co-morbidities in an Australian cohort of total hip arthroplasty (THA) and total knee arthroplasty (TKA) patients using two diagnoses based co-morbidity measures (Charlson [17] and Elixhauser [18]) and one prescription based measure (RxRisk-V [19, 22]) and (2) compare the agreement of individual co-morbidities amongst the measures.

## Methods

### Study design, setting, and sample

A cross-sectional study was conducted on a cohort of THA and TKA patients, who had their procedures between 2001 and 2012. Patients receiving care subsidised by the Australian Government Department of Veterans’ Affairs (DVA) were included in the study. De-identified administrative inpatient encounter information and prescription medicine (inpatient and outpatient) data for this captured population was used.

The study sample included patients aged ≥18 years old, who had all health services subsidised by DVA, and underwent elective primary unilateral THA procedures identified using International Classification of Disease, 10^th^ Revision, Australian Modification (ICD-10-AM) code 4931800 and elective primary unilateral TKA procedures identified using ICD-10-AM codes 4951800, 4952100, 4952102, 4952400.

### Co-morbidity measures and data sources

The RxRisk-V [19] is a co-morbidity prescription based measure that uses patients’ medication histories to determine the presence of 45 conditions [22]. This measure has been shown to be predictive of cost of care [19, 22] and mortality [23–25] in different patient samples using both inpatient and outpatient pharmacy data [23, 25]. In this study a modified RxRisk-V was used with 42 conditions; the conditions ostomy, neurogenic bladder, and urinary incontinence were excluded.

The Charlson co-morbidity measure typically uses inpatient hospitalisations over a specified time period to identify the presence of 17 conditions and calculate an overall comorbidity score [21, 26]. The Charlson score was originally developed to predict mortality and assist with case mix adjustment in regards to this outcome, but has been applied to several other outcomes now, including some surgical outcomes [16, 27].

The Elixhauser co-morbidity measure also typically uses inpatient hospitalisations during a specific period to calculate co-morbidities. The most common form of this measure identifies the presence of 30 conditions and has been evaluated as a predictor of blood transfusions, length of stay, and mortality [26, 28]. This measure was developed by the Agency for Healthcare Research and Quality (AHRQ) Healthcare Cost and Utilisation Project and is widely used in health services research [18, 29].

The RxRisk-V and Charlson have 6 common conditions, the Elixhauser and RxRisk-V have 10 common conditions, and the Charlson and Elixhauser have 12.

Using the DVA administrative database all inpatient hospitalisations and prescription medicine history were identified for the study sample. The database contains details of all prescription medications, medical, allied health services and hospitalisations provided to veterans for which DVA pays a subsidy. In the dataset, medications are coded according to the World Health Organization Anatomic, Therapeutic and Chemical Classification, and the Pharmaceutical Benefits Schedule item codes. Hospitalisations are coded according to the ICD-10-AM. DVA also maintains a client file, which contains information on gender, date of birth, date of death, and family status for a treatment population that in September 2011 was 242,000 people.

In this study, the 12 month period preceding the discharge date of the arthroplasty procedure was used to ascertain the co-morbidities according to the two diagnoses based co-morbidity measures (Charlson and Elixhauser) using DVA hospital records. The arthroplasty procedure hospitalisation was included in the calculation of the diagnostic co-morbidity measures. The 12 month prescription dispensing history preceding the admission date for the arthroplasty procedure was utilised to measure RxRisk-V.

### Statistical analysis

Frequencies, proportions, means, standard deviations (SD), medians and interquartile ranges (IRQs) were used to describe the sample. Prevalence of co-morbidities by each measure was calculated. Agreement between specific co-morbidity indicators between measures (where conditions were common) was calculated using Kappa (κ) statistics and 95 % confidence intervals (CI). κ agreement thresholds used were: slight: 0.01 ≤ κ ≤ 0.20, fair: 0.21 ≤ κ ≤ 0.40, moderate:0.41 ≤ κ ≤0.60, substantial: 0.61 ≤ κ ≤ 0.80, almost perfect: 0.80 ≤ κ ≤ 0.99, perfect: κ =1.0 [30]. SAS 9.4 (SAS Institute, Cary, NC, USA) was used for all analyses.

This study has ethics approval from the Australian DVA and University of South Australia human research ethics committees. The ethics committees also waived the requirement for informed consent.

## Results

During the study period 11,848 patients underwent THA and 18,972 TKAs. Both cohorts had a slightly higher proportion of males (50.2 % THA and 52.3 % TKA) than females and procedures were mostly performed in private hospitals (95.6 % THA and 96.6 % TKA). The median age was 80.9 (IQR 76.4-84.4) years old for patients with THAs and 79.8 (IQR = 74.7-83.5) years old for patients with TKAs. See Table 1 for sample details.

**Table 1:** Total hip and knee arthroplasty patients characteristics, 2001–2012

		Total hip arthroplasty		Total knee arthroplasty	
		N	(%)	N	(%)
Total		11,848	100.0	18,972	100.0
Gender	Females	5,898	49.8	9,047	47.7
	Males	5,950	50.2	9,925	52.3
Age, years (median, IQR)		80.9	76.4–84.4	79.8	74.7–83.5
THA diagnoses (ICD-10-AM code)	Other primary coxarthrosis (M161)	9,648	81.4	-	-
	Coxarthrosis unspecified (M169)	1,109	9.4	-	-
	Unspecified osteonecrosis pelvis thigh (M8795)	343	2.9	-	-
	Other	748	6.3		
TKA Diagnosis (ICD-10-AM Code)	Other primary gonarthrosis (M171)	-	-	16,329	86.1
	Gonarthrosis unspecified (M179)	-	-	1,437	7.6
	Primary gonarthrosis bilateral (M170)	-	-	489	2.6
	Other	-	-	717	3.8
Number of RxRisk-V co-morbidities	0	1,466	12.4	3,290	17.3
	1–2	747	6.3	938	4.9
	3–4	2,041	17.2	2,785	14.7
	5–6	2,888	24.4	4,398	23.2
	≥7	4,706	39.7	7,561	39.9
Number of Elixhauser co-morbidities	0	6,087	51.4	9,910	52.2
	1–2	4,333	12.1	7,034	37.1
	≥3	1,428	12.1	2,028	10.7
Number of Charlson co-morbidities	0	8,529	72.0	13,917	73.4
	1–2	2,946	24.9	4,628	24.4
	≥3	373	3.1	427	2.3

*IQR* interquartile range, *THA* total hip arthroplasty, *TKA* total knee arthroplasty, *ICD-10-AM* International Classifications of Disease, 10th Revision, Australian Modification

The mean number of RxRisk-V, Elixhauser, and Charlson co-morbidities in the THA (5.5 (SD = 3.3), 0.9 (SD = 1.3), 0.4 (SD = 0.8), respectively) and TKA (5.4 (SD = 3.5), 0.9 (SD = 1.2), 0.4 (SD = 0.7), respectively) cohorts was similar. The three most common co-morbidities identified by the RxRisk-V were also similar between the THA and TKA cohort, and included: the musculoskeletal conditions of pain treated with anti-inflammatories (58.7 % THA, 55.9 % TKA) and pain treated with narcotics (55.0 % THA, 50.9 % TKA), and cardiovascular diseases that involved treatment with anticoagulation agents (52.6 % THA, 48.4 % TKA). Using the Elixhauser measure, the three most prevalent co-morbidities in patients having a THA were: hypertension (22.0 %), arrhythmias (14.7 %), and fluid and electrolyte disorders (7.5 %); in patients with TKAs the conditions were: hypertension (23.4 %), arrhythmias (13.5 %), and diabetes with chronic complications (7.7 %). Using the Charlson measure the three more prevalent co-morbidities of THA and TKA patients were: uncomplicated diabetes (7.1 % THA, 8.1 % TKA), diabetes with chronic complications (6.3 % THA, 7.3 % TKA), and chronic pulmonary disease (5.5 % THA, 4.5 % TKA). See Table 2 for co-morbidities by the three measures and overall prevalence of conditions.

**Table 2:** Mean total scores and prevalence of individual conditions by each co-morbidity measure and total computation

	Total hip arthroplasty (*N* = 11,848)				Total knee arthroplasty (*N* = 18,972)			
	RxRisk-V	Elixhauser	Charlson	Total	RxRisk-V	Elixhauser	Charlson	Total
Mean score (SD)	5.5 (3.3)	0.9 (1.3)	0.4 (0.8)	6.2 (3.6)	5.4 (3.5)	0.9 (1.2)	0.4 (0.7)	6.1 (3.7)
	N (%)	N (%)	N (%)	N (%)	N (%)	N (%)	N (%)	N (%)
Cancer								
Lymphoma	-	42 (0.4)	-	42 (0.4)	-	33 (0.2)	-	33 (0.2)
Malignancies	423 (3.6)		257 (2.2)	648 (5.5)	757 (4.0)		285 (1.5)	1,004 (5.3)
Metastatic cancer	-	60 (0.5)	60 (0.5)	60 (0.5)	-	43 (0.2)	43 (0.2)	43 (0.2)
Solid tumor without metastatis	-	303 (2.6)	-	306 (2.6)	-	398 (2.1)	-	398 (2.1)
Cardiovascular/blood								
Anticoagulation agents/coagulopathy	6,230 (52.6)	130 (1.1)	-	6,274 (53.0)	9,177 (48.4)	168 (0.9)	-	9,227 (48.6)
Antiplatelets agents	3,975 (33.5)	-	-	3,975 (33.5)	6,055 (31.9)	-	-	6,055 (31.9)
Arrhythmias	1,206 (10.2)	1,744 (14.7)	-	2,313 (19.5)	1,766 (9.3)	2,561 (13.5)	-	3,427 (18.1)
Cerebrovascular disease	-	-	273 (2.3)	273 (0.3)	-	-	353 (1.9)	353 (1.8)
Congestive heart failure	1,454 (12.3)	471 (4.0)	471 (4.0)	1,636 (13.8)	2,142 (11.3)	608 (3.2)	608 (3.2)	2,452 (12.9)
Hyperlipidaemia	4,377 (36.9)	-	-	4,377 (36.9)	7,115 (37.5)	-	-	7,115 (37.5)
Hypertension^a^	5,644 (47.6)	2,612 (22.0)	-	6,638 (56.0)	8,866 (46.7)	4,433 (23.4)	-	10,629 (56.0)
Ischemic heart disease/angina	1,375 (11.6)	-	-	1,375 (11.6)	2,054 (10.8)	-	-	2,054 (10.8)
Ischemic heart disease/hypertension	4,054 (34.2)	-	-	4,054 (34.2)	6,329 (33.4)	-	-	6,329 (33.4)
Myocardial infarction	-	-	333 (2.8)	333 (2.8)	-	-	410 (2.2)	410 (2.2)
Peripheral vascular disease	-	299 (2.5)	299 (2.5)	299 (2.5)	-	346 (1.8)	346 (1.8)	346 (1.8)
Pulmonary circulation disorders	-	136 (1.1)	-	136 (1.2)	-	288 (1.5)	-	288 (1.5)
Valvular disease	-	308 (2.6)	-	308 (2.6)	-	414 (2.2)	-	414 (2.2)
Endocrine								
Diabetes (uncomplicated)	905 (7.6)	755 (6.4)	838 (7.1)	1,271 (10.7)	1,782 (9.4)	1,405 (7.4)	1,530 (8.1)	2,423 (12.8)
Diabetes (complicated)	-	794 (6.7)	747 (6.3)	794 (6.7)	-	1,452 (7.7)	1387 (7.3)	1,452 (7.7)
Hypothyroidism	788 (6.7)	67 (0.6)	-	801 (6.8)	1339 (7.1)	95 (0.5)	-	1,369 (7.2)
Pancreatic insufficiency	20 (0.2)	-	-	20 (0.2)	31 (0.2)	-	-	31 (0.2)
Gastrointestinal								
Gastric acid disorder	5,307 (44.8)	-	-	5,307 (44.8)	8,436 (44.5)	-	-	8,436 (44.5)
Inflammatory bowel syndrome	118 (1.0)	-	-	118 (1.0)	180 (1.0)	-	-	180 (1.0)
Hepatitis C	0 (0.0)	-	-	0 (0.0)	0 (0.0)	-	-	0 (0.0)
Liver disease (mild)	-	25 (0.2)	26 (0.2)	33 (0.3)	-	42 (0.2)	47 (0.2)	55 (0.3)
Liver disease (severe) or failure	479 (4.0)	-	11 (0.1)	487 (4.1)	542 (2.9)	-	9 (<0.01)	551 (2.9)
Peptic ulcer disease	-	83 (0.7)	119 (1.0)	119 (1.0)	-	102 (0.5)	155 (0.8)	155 (0.8)
Muscuoskeletal/pain related								
Gout	1,187 (10.0)	-	-	1,187 (10.0)	2,210 (11.6)	-	-	2,210 (11.7)
Migraine	37 (0.3)	-	-	37 (0.3)	104 (0.5)	-	-	104 (0.6)
Osteoporosis/Pagets	1,463 (12.3)	-	-	1,463 (12.3)	2,017 (10.6)	-	-	2,017 (10.6)
Pain	6,512 (55.0)	-	-	6,512 (55.0)	9,663 (50.9)	-	-	9,663 (50.9)
Pain/Inflammation	6,958 (58.7)	-	-	6,958 (58.7)	10,611 (55.9)	-	-	10,611 (55.9)
RA/collage vascular disorders	-	197 (1.7)	176 (1.5)	197 (1.7)	-	348 (1.8)	320 (1.7)	349 (1.8)
Neurologic								
Dementia	92 (0.8)	-	209 (1.8)	249 (2.1)	94 (0.5)	-	195 (1.0)	237 (1.3)
Epilepsy	535 (4.5)	-	-	535 (4.5)	838 (4.4)	-	-	838 (4.4)
Paralysis (or Paraplegia/Hemiplegia)	-	86 (0.7)	86 (0.7)	86 (0.7)	-	112 (0.6)	112 (0.6)	112 (0.6)
Parkinson’s disease	198 (1.7)	-	-	198 (1.7)	367 (1.9)	-	-	367 (1.9)
Other neurological disorders	-	155 (1.3)	-	155 (1.3)	-	205 (1.1)	-	205 (1.1)
Nutritional/obesity								
Blood loss anaemia	-	84 (0.7)	-	84 (0.7)	-	67 (0.4)	-	67 (0.4)
Deficiency anaemia	-	203 (1.7)	-	203 (1.7)	-	223 (1.2)	-	223 (1.2)
Fluid and electrolyte disorders	-	890 (7.5)	-	890 (7.5)	-	1,095 (5.8)	-	1,095 (5.8)
Hyperkalaemia	8 (0.1)	-	-	8 (0.1)	18 (0.1)	-	-	18 (0.1)
Malnutrition	22 (0.2)	-	-	22 (0.2)	37 (0.2)	-	-	37 (0.2)
Obesity	-	194 (1.6)	-	194 (1.6)	-	500 (2.6)	-	500 (2.6)
Weight loss	-	123 (1.0)	-	123 (1.0)	-	99 (0.5)	-	99 (0.5)
Psychological/behavioural								
Alcohol abuse/dependence	11 (0.1)	113 (1.0)	-	120 (1.0)	27 (0.1)	177 (0.9)	-	193 (1.0)
Anxiety and tension	1,459 (12.3)	-	-	1,459 (12.3)	2,344 (12.3)	-	-	2,340 (12.3)
Bipolar disorder	30 (0.3)	-	-	30 (0.3)	49 (0.3)	-	-	49 (0.3)
Depression	2,598 (21.9)	213 (1.8)	-	2,667 (22.5)	4,484 (23.6)	293 (1.5)	-	4,567 (24.1)
Drug abuse		14 (0.1)	-	14 (0.1)		20 (0.1)	-	20 (0.1)
Psychotic illness/psychoses	250 (2.1)	16 (0.1)	-	259 (2.2)	391 (2.1)	22 (0.1)	-	404 (2.1)
Smoking cessation	102 (0.9)	-	-	102 (0.9)	159 (0.8)	-	-	159 (0.8)
Renal/urologic								
Benign prostatic hypertrophy	445 (3.8)	-	-	445 (3.8)	731 (3.9)	-	-	731 (3.9)
Renal disease/failure	162 (1.4)	436 (3.7)	388 (3.3)	560 (4.7)	290 (1.5)	575 (3.0)	524 (2.8)	826 (4.4)
Respiratory								
Chronic pulmonary disease	-	652 (5.5)	651 (5.5)	652 (5.5)	-	858 (4.5)	856 (4.5)	858 (4.5)
Reactive airway disease	2,461 (20.8)	-	-	2,461 (20.8)	3,815 (20.1)	-	-	3,815 (20.1)
Tuberculosis	0 (0.0)	-	-	0 (0.0)	1 (<0.01)	-	-	1 (<0.01)
Miscellaneous								
Allergies	1,660 (14.0)	-	-	1,660 (14.0)	,3046 (16.1)	-	-	3,046 (16.1)
Glaucoma	1,091 (9.2)	-	-	1,091 (9.2)	1,642 (8.7)	-	-	1,642 (8.7)
HIV/AIDS	0 (0.0)	0 (0.0)	0 (0.0)	0 (0.0)	0 (0.0)	0 (0.0)	0 (0.0)	0 (0.0)
Psoriasis	63 (0.5)	-	-	63 (0.5)	103 (0.5)	-	-	103 (0.5)
Steroid-responsive conditions	1,889 (15.9)	-	-	1,889 (15.9)	3,345 (17.6)	-	-	3,345 (17.6)
Transplant	3 (<0.01)	-	-	3 (<0.01)	3 (<0.01)	-	-	3 (<0.01)

*SD* standard deviation, *CI* confidence interval, *RA* rheumatoid arthritis, *HIV/AIDS* human immunodeficiency virus/Acquired immune deficiency virus

^a^Complicated and uncomplicated combined

Combining all measures, 64 unique co-morbidities were identified, of these 28 were only identified using the RxRisk-V, 11 using the Elixhauser, and 2 using the Charlson. The most prevalent co-morbidities only identified by the RxRisk-V included: pain treated with anti-inflammatories, pain treated with narcotics, gastric acid disorder, hyperlipidaemia, ischemic heart disease (both in combination with angina and hypertension), diseases treated with antiplatelets agents, and reactive airway disease. The most prevalent co-morbidities only identified by the Elixhauser included fluid and electrolyte disorders, obesity, valvular disease, history of solid tumour, deficiency anaemia, and obesity. The co-morbidities only identified by Charlson were cerebrovascular disease and myocardial infarction. See Table 2 other co-morbidities specific to each measure.

There was a relationship between co-morbidity measures for common indicators (Table 3). RxRisk-V and the Elixhauser, diabetes was the only co-morbidity with substantial agreement between the two measures (κ = 0.63, 95 % CI 0.60-0.65 for THA, κ = 0.61, 95 % CI 0.59-0.63 for TKA). Similarly, when comparing the RxRisk-V and Charlson measures, diabetes was also the only co-morbidity with substantial agreement ((κ = 0.63, 95 % CI 0.60-0.65 for THAs, κ = 0.61, 95 % CI 0.59-0.63 for TKAs). When comparing the two diagnostic based measures, the Charlson and Elixhauser, for conditions that did not have identical coding, the agreement was almost perfect for uncomplicated diabetes, diabetes with chronic complications, peptic ulcer disease, rheumatoid arthritis, and renal failure. Mild liver disease had substantial agreement between the Charlson and Elixhauser (κ = 0.71, 95 % CI 0.56-0.85 for THA and κ = 0.76, 95 % CI 0.67-0.86 for TKA).

**Table 3:** Agreement between each co-morbidity measure of individual conditions. Cohen’s Kappa coefficient and 95 % confidence intervals for agreement estimations

	Total hip arthroplasty			Total knee arthroplasty		
	RxRisk-V Elixhauser^a^	RxRisk-V Charlson^b^	Elixhauser Charlson^c^	RxRisk-V Elixhauser^a^	RxRisk-V Charlson^b^	Elixhauser Charlson^c^
	ƙ (95 % CI)	ƙ (95 % CI)	ƙ (95 % CI)	ƙ (95 % CI)	ƙ (95 % CI)	ƙ (95 % CI)
Cancer						
Malignancies	-	0.07 (0.04-0.10)	-	-	0.06 (0.03-0.08)	-
Metastatic cancer		-	1.00 (1.00-1.00)		-	1.00 (1.00-1.00)
Cardiovascular/blood						
Anticoagulation agents/coagulopathy	0.01 (0.00-0.01)			0.00 (0.00-0.01)		
Arrhythmias	0.35 (0.33-0.38)	-	-	0.34 (0.32-0.36)	-	-
Congestive heart failure	0.26 (0.23-0.28)	0.26 (0.23-0.28)	1.00 (1.00-1.00)	0.18 (0.16-0.20)	0.18 (0.16-0.20)	1.0 (1.00-1.00)
Hypertension (complicated and uncomplicated)	0.13 (0.11-0.14)	-	-	0.13 (0.11-0.14)	-	-
Peripheral vascular disease	-	-	1.00 (1.00-1.00)	-	-	1.00 (1.00-1.00)
Endocrine						
Diabetes (uncomplicated)	0.63 (0.60-0.65)	0.63 (0.60-0.65)	0.94 (0.93-0.96)	0.61 (0.59-0.63)	0.61 (0.59-0.63)	0.95 (0.95-0.96)
Diabetes (complicated)	-	-	0.97 (0.96-0.98)	-	-	0.98 (0.97-0.98)
Hypothyroidism	0.12 (0.09-0.15)	-	-	0.08 (0.06-0.10)	-	-
Gastrointestinal						
Liver disease (mild)	-		0.71 (0.56-0.85)	-	-	0.76 (0.67-0.86)
Liver disease (severe) or failure	-	0.01 (0.00-0.02)	-	-	0.00 (0.00-0.00)	-
Peptic ulcer disease	-	-	0.82 (0.76-0.88)	-	-	0.79 (0.74-0.85)
Muscuoskeletal/pain related						
Rheumatoid arthritis/collage vascular disorders	-	-	0.94 (0.92-0.97)	-	-	0.95 (0.94-0.97)
Neurologic						
Dementia	-	0.34 (0.27-0.41)	-	-	0.36 (0.28-4.43)	-
Paralysis (or Paraplegia/Hemiplegia)	-	-	1.00 (1.00-1.00)	-	-	1.00 (1.00-1.00)
Psychological/behavioral						
Alcohol abuse/dependence	0.06 (0.00-0.12)	-	-	0.10 (0.05-0.16)	-	-
Depression	0.07 (0.06-0.08)	-		0.06 (0.05-0.07)	-	
Psychotic illness/psychoses	0.05 (0.01-0.09)	-		0.04 (0.01-0.07)	-	
Renal/urologic						
Renal disease/failure	0.11 (0.07-0.15)	0.11 (0.08-0.15)	0.94 (0.92-0.96)	0.07 (0.04-0.10)	0.07 (0.05-0.10)	0.95 (0.94-0.97)
Respiratory						
Chronic pulmonary disease	-	-	1.00 (1.00-1.00)	-	-	1.00 (1.00-1.00)

^a^RxRisk-V and Elixhauser have ten conditions in common

^b^RxRisk-V and Charlson have six conditions in common

^c^Elixhauser and Charlson have 12 conditions in common, six where the diagnoses codes are identical (only 11/5 are shown because no cases of HIV/AIDS were identified in this sample)

## Discussion

The co-morbidity measures evaluated in this study yielded a different prevalence of co-morbidities and a wider variation of agreement between common conditions. In our sample of joint arthroplasty patients, the most common non-musculoskeletal related co-morbidities were hypertension, disorders that required anticoagulation agents, and gastric acid disorders. We also found that except for diabetes, which had good agreement between all three co-morbidity measures, there was low agreement between the prescription based RxRisk-V and the diagnoses based Elixhauser and Charlson co-morbidity measures for common conditions. As expected, between the diagnoses based measures there was substantial agreement between common co-morbidities.

The prevalence of co-morbidities identified using all three measurements had some commonalties and differences with previous estimates in large cohorts of joint arthroplasty patients. Some of the differences are attributed to the method of co-morbidity ascertainment in different studies. The Elixhauser was the more commonly used measure in the published literature, such as Cram et al.’s US Medicare studies [3, 4], Kapoor et al.’s US DVA study [31], the Kaiser Permanente Total Joint Replacement registry studies [32, 33], and the US California and New York states cohort studies by Dy et al. [34, 35]. But other studies have used different measures, such as an US Medicare TKA study that used a combination of the Charlson and Elixhauser [5], and a Finish arthroplasty registry study that used a non validated measure inclusive of diagnostic codes, medication prescriptions, and drug reimbursement for certain conditions [36]. Most studies reported similar high prevalence of hypertension (range 43–70 %) [5, 31–35], but the Finish study reported a lower prevalence (17.7 % in THA and 20.8 % in TKA) than these cohorts and ours [36]. The prevalence of diabetes was also similar between our cohort (approximately 10–13 %) and those of the US California and New York states studies (9 % in the THAs and 14 % in TKAs), US Medicare THA cohort (10-15 % recent estimates) and the Finish arthroplasty registry cohort (5.5–7.5 %) [3, 35, 36]. This prevalence was lower, however, than those reported by other US cohorts (Kaiser Permanente registry 18–26 %, Medicare TKA patients 22 %, and DVA patients 26.5 %) [4, 5, 31–33]. The Kaiser Permanente registry ascertained diabetes using the institution’s diabetes registry, instead of the Elixhauser, which could contribute to the higher prevalence reported [37]. The prevalence of congestive heart failure (approximately 13 %) was also similar to that reported by one study reporting on the US TKA Medicare cohort (10 %) [6], but higher than all other studies reviewed (range 2.6–5.2 %) [3, 4, 31–36]. Conversely, the prevalence of chronic obstructive pulmonary disease in our sample (5–6 %) was lower than that reported by almost all cohorts (range 11–18 %) [6, 31, 34, 35] but the Finish arthroplasty register cohort (6–8 %) [36]. While there are obvious differences in the cohorts of patients in these studies, disease prevalence differences are also due to how they were identified- highlighting the need for consideration in how co-morbidities are determined.

There was only a strong agreement between the co-morbidities identified by the Risk-V and the Elixhauser (10 common conditions) and Charlson (6 common conditions) for the condition of diabetes. For all other conditions, the agreement was weak. This agrees with previous comparison of the Charlson and RxRisk-V in a cohort of older non-joint arthroplasty Australian veterans [38]. Because of this lack of agreement amongst measurements, it is necessary to understand what measurements are used by different studies. A study using only diagnoses based measures to identify congestive heart failure, hypertension, or depression will underestimate these diagnoses, while a study using only RxRisk-V will underestimate renal disease/failure, dementia, and alcohol abuse. Underestimating these co-morbidities can impact study estimates. Utilizing validated measures is also recommended to insure that comparisons with other study results are possible.

In addition to the differences in prevalence estimates of co-morbidities, there are further considerations for choosing the appropriate measure for co-morbidity ascertainment. Specifically, within the 64 co-morbidities identified by all measures used in this study, 28 were only identified by the RxRisk-V, 11 only by the Elixhauser and two only by the Charlson. Studies requiring the nutritional/obesity related co-morbidities such as obesity, blood loss anaemia, deficiency anaemia, fluid and electrolyte disorders, and weight loss should use the Elixhauser measure. Studies that require detail on the specific cardiovascular disease a patient is actually being treated for (e.g. anticoagulation disorder, hyperlipidaemia, ischemic heart disease/angina, and ischemic heart disease/hypertension) should use the RxRisk-V for obtaining this information. The Charlson measure should be used if history of myocardial infarction or cerebrovascular disease are needed. If a more comprehensive understanding of a patients’ co-morbidity profile is necessary, we suggest using all the co-morbidity indices for both the inclusion of a greater number of conditions and a likely greater sensitivity in identifying certain conditions and provide greater amount of information to conduct case mix adjustment.

This study has several limitations. Information bias due to our use of administrative data, which can suffer from coding errors, missing data, linkage problems, and lack of detailed clinical information, was possible. Additionally, because of our sampling frame the prevalence of co-morbidities in our study may not be representative of the greater population undergoing joint arthroplasty in Australia or in other countries, where the median age of TKA and THA cohorts is between 65 and 70 years old. Ours is a sample of patients who are members of the Australian DVA system, an older patient population due to how their benefits are granted. Due to their older age we would expect them to have more co-morbidities than younger arthroplasty cohorts. However, they are representative of an increasingly greater number of patients undergoing joint arthroplasty later in life and therefore offer valuable information in regards to this specific demographic.

Our study strengths included the utilisation of a captive membership population with a comprehensive database of prescription medications dispensed to its members. Due to the nature of DVA services payments, all the hospitalisation and prescriptions our cohort of patients obtains within Australia is captured. A further strength of our study is that a previous validation study has shown acceptable results in using the RxRisk-V in the Australian population in identifying co-morbidities as compared to self-reported conditions [25]. Finally, all patients in our study cohort have unique identifiers, minimizing the likelihood of data handling bias when linking their hospitalisation, demographic, and hospitalisation information.

## Conclusion

Co-morbidity measures allow us to efficiently evaluate the disease burden of large cohorts of patients using existing data, such as administrative encounter and pharmacy dispensing records. Our study, along with others [38, 39] shows that the prescription based RxRisk-V measure and diagnostic based Charlson and Elixhauser measures identify a different prevalence of disease for the same conditions and have little agreement amongst them (with the exception of diabetes). Some conditions were better detected using prescription medication monitoring, while others were detected using previously inputted diagnostic codes. The specific co-morbidity measure should be chosen based on conditions necessary for that particular study, the acceptable or desirable degree of sensitivity or specificity in identifying these co-morbidities, and with the understanding of limitations involved with each of the specific measures.

## Abbreviations

AHRQ: Agency for Healthcare Research and Quality
DVA: Department of Veterans’ Affairs
ICD-10-AM: International Classification of Disease, 10^th^ Revision, Australian Modification
IRQ: interquartile ranges
SD: standard deviations
THA: total hip arthroplasty
TKA: total knee arthroplasty
US: United States
Κ: kappa
